# Electromyographic Patterns of Scapular Muscles During Four Variations of Protraction–Retraction Exercises

**DOI:** 10.3390/life15121840

**Published:** 2025-11-29

**Authors:** Eui-Young Jung, Su-Yeon Roh, Woo-Lim Mun

**Affiliations:** 1 Department of Health Science, Gachon University Graduate School, Incheon 21936, Republic of Korea; noel950@gachon.ac.kr; 2 Department of Exercise Rehabilitation, Gachon University, Incheon 21936, Republic of Korea

**Keywords:** scapular dyskinesis, scapular, electromyography, muscle activation, push-up plus, serratus anterior

## Abstract

(1) Background: How variations of the push-up plus (PUP)—particularly changes in the base of support and scapular excursion—affect scapular muscle activation remains unclear. This study compared phase-specific electromyographic (EMG) activity of scapular muscles during four protraction–retraction exercises. (2) Methods: Twenty-six healthy male participants (age: 22.88 ± 1.45 years; height: 1.74 ± 0.05 m; weight: 77.31 ± 8.61 kg; body mass index (BMI): 25.61 ± 2.43 kg/m^2^) with Pilates experience performed four scapular protraction–retraction exercises under two base-of-support (quadruped vs. single-leg) and two movement-range (PUP vs. STD) conditions. Exercise order was randomized, and sufficient rest intervals were provided to minimize fatigue effects. Surface electromyography was recorded from six scapular muscles and normalized to maximal voluntary isometric contraction. The study was registered on CRIS (KCT0010032). (3) Results: Single-leg PUP showed the greatest serratus anterior (SA) activation, with increases of approximately 30% in protraction, 20–25% in isometric, and 15–20% in retraction. STD variations elicited higher trapezius activation, especially during large scapular excursions. The UT/SA ratio was significantly lower in PUP conditions (η^2^_p_ = 0.544), reflecting a more favorable stabilization pattern. (4) Conclusions: This experimental repeated-measures study demonstrates that combining single-leg support with traditional PUP meaningfully increases SA recruitment across all phases, whereas increased scapular range enhances trapezius engagement. These findings provide novel phase-specific insights into how PUP variations modulate closed-chain scapular stabilization and may assist clinicians in selecting targeted exercises. Interpretation should be limited to trained healthy males.

## 1. Introduction

Scapular stability plays a crucial role in maintaining optimal shoulder function and preventing injuries. Effective shoulder mechanics rely on the scapula serving as both a stable base and a structure capable of proper motion [1,2,3]. Proper scapular control not only supports efficient shoulder function but also helps prevent shoulder overuse injuries and reduce the incidence of instability or rotator cuff overload [4]. Dysfunction of scapular movement patterns, commonly referred to as scapular dyskinesis, has been linked to various shoulder pathologies, including impingement syndrome, rotator cuff tears, glenohumeral instability, and scapular winging [5,6,7]. Therefore, developing effective exercise interventions targeting scapular stabilization has become a critical component of shoulder rehabilitation and injury prevention programs [8,9].

One of the commonly prescribed scapular stabilization exercises is the push-up plus (PUP), which effectively activates the serratus anterior (SA) and improves scapular control [10,11]. The SA and trapezius play essential roles in maintaining scapular mobility and stability during upper-limb movements, and dysfunction of the SA has been linked to insufficient upward rotation, excessive anterior tilt, impingement, and scapular winging [12,13,14,15,16]. Because SA weakness often leads to compensatory overactivation of the upper trapezius, levator scapulae, or pectoralis major, while the middle and lower trapezius act synergistically with the SA to support upward rotation and posterior tilt, analyzing their activation patterns together is crucial for understanding compensatory strategies. Accordingly, EMG-based assessments of SA and its synergists/antagonists—including activation ratios such as UT/SA or UT/LT—provide meaningful information about the efficiency of scapular stabilization during exercise [17,18,19].

In addition, the single-leg condition may increase postural demands by reducing the base of support, which could challenge the neuromuscular control of the scapulothoracic complex [20]. Moreover, performing scapular stabilization tasks under a single-leg condition introduces not only a reduced base of support but also a rotational stability challenge. This asymmetric loading requires greater coordination between the trunk and scapular stabilizers to maintain body alignment, potentially influencing scapular muscle activation [21,22]. Previous studies using quadruped or four-point kneeling postures have shown that lifting one limb significantly elevates trunk and hip stabilizer activation to maintain lumbopelvic stability [23]. These findings suggest that unilateral support tasks tend to heighten postural control requirements, which may, in turn, influence the activation of scapular stabilizing muscles during closed-chain shoulder exercises.

Electromyography (EMG) is widely used to assess muscle activation patterns, yet few studies have examined how asymmetric or unstable postural conditions influence scapular activation [24,25,26]. Most PUP research has focused on hand position, shoulder width, or support surface [11,27], while the effects of altering the range and direction of scapular motion—particularly comparing the traditional PUP with the sternum-drop variation—remain unclear. Reported SA activation varies widely (40–80% MVIC), likely because prior analyses evaluated only overall EMG activity [28,29,30,31]. In this study, the sternum drop was included to isolate the effect of maximal retraction before protraction and to examine end-range control demands not addressed previously. Phase-specific EMG analysis (isometric, protraction, retraction) may provide clearer insight into stabilization mechanisms. Although greater scapular excursion is known to increase muscle activation [32,33], no studies have investigated PUP performed with maximal range of motion. These variations differ in scapular displacement and neuromuscular demands, offering useful information for optimizing closed-chain stabilization exercises.

Therefore, the purpose of this study was to investigate scapular muscle activation patterns during four variations of scapular protraction–retraction exercises. We hypothesized that single-leg variations would increase serratus anterior activation, while increased scapular excursion would preferentially enhance trapezius recruitment compared with the conventional PUP.

## 2. Methods

### 2.1. Study Design

This study was designed as a cross-sectional and repeated-measures within-group comparison. All sessions were performed between October and December 2024 in the laboratory of Gachon University, Incheon, South Korea. The study was approved by the Gachon University Institutional Review Board (No. 044396-202409-HR-152-01) and registered on CRIS (No. KCT0010032). Written informed consent was obtained from all participants before their enrollment in the study.

### 2.2. Participants

Participants were recruited via announcements posted on university bulletin boards and social networking services using Facebook and Instagram. The inclusion criteria were as follows: (1) healthy male aged between 20 and 30 years, and (2) individuals with at least 6 months of Pilates exercise experience. All participants were recreationally active and engaged in regular physical activity at least two times per week, including resistance or Pilates-based exercise. Participants with prior Pilates experience were recruited to ensure consistent performance and reliable EMG data. This criterion minimized movement errors and variability in muscle activation caused by lack of stabilization control. The exclusion criteria were as follows: (1) a diagnosis of orthopedic or neurological disorders, (2) having any cardiovascular, pulmonary, or other medical conditions that could limit exercise. The sample size estimation was calculated using G*Power 3.1.9.7 (Heinrich Heine University, Düsseldorf, Germany) with the following parameters: effect size f = 0.25, alpha error probability = 0.05, power = 0.80, number of groups = 1, number of measurements = 4 [34]. The calculated sample size was 24, and considering a 10% dropout rate, a total of 27 subjects were recruited. One participant was unable to participate because he did not meet the criteria due to his orthopedic history. Therefore, twenty-six healthy males (age: 22.88 ± 1.45 years; height: 1.74 ± 0.05 m; weight: 77.31 ± 8.61 kg; skeletal muscle: 36.06 ± 3.41 kg; body fat rate: 16.9 ± 5.2%; body mass index (BMI): 25.61 ± 2.43 kg/m^2^; right dominant leg: 22; left dominant leg: 4) participated in the current study. The dominant side was defined as the side of the hand primarily used for writing and daily tasks. During the single-leg condition, participants maintained an ipsilateral configuration (dominant arm and dominant leg on the same side) to minimize trunk rotation and ensure consistent loading between participants [35,36].

### 2.3. Experimental Procedures

All procedures were carried out in the Gachon University laboratory, and all participants visited the laboratory twice at seven-day intervals. During the first visit, anthropometric measurements were obtained, and participants were familiarized with the exercise protocol, including EMG electrode placement and movement practice, to minimize learning effects. Anthropometric variables, including height, weight, body mass index (BMI), body fat percentage, and skeletal muscle mass, were measured using a body composition analyzer (InBody BMS330, Biospace Co., Ltd., Seoul, Republic of Korea). The familiarization process involved detailed instruction and practice of the exercise movements until participants were able to perform them accurately. At the second visit, maximal voluntary isometric contraction (MVIC) was assessed, and electromyography (EMG) data were collected for the four exercises [37]. Before the second session, all participants confirmed that they were free of muscle soreness or fatigue. To minimize the order effect, the order of exercises was randomized using a web-based permutation generator (dcode.fr). Each exercise was repeated three times, with a five-minute rest period between each session.

### 2.4. Exercise Protocol

**Figure 1:**
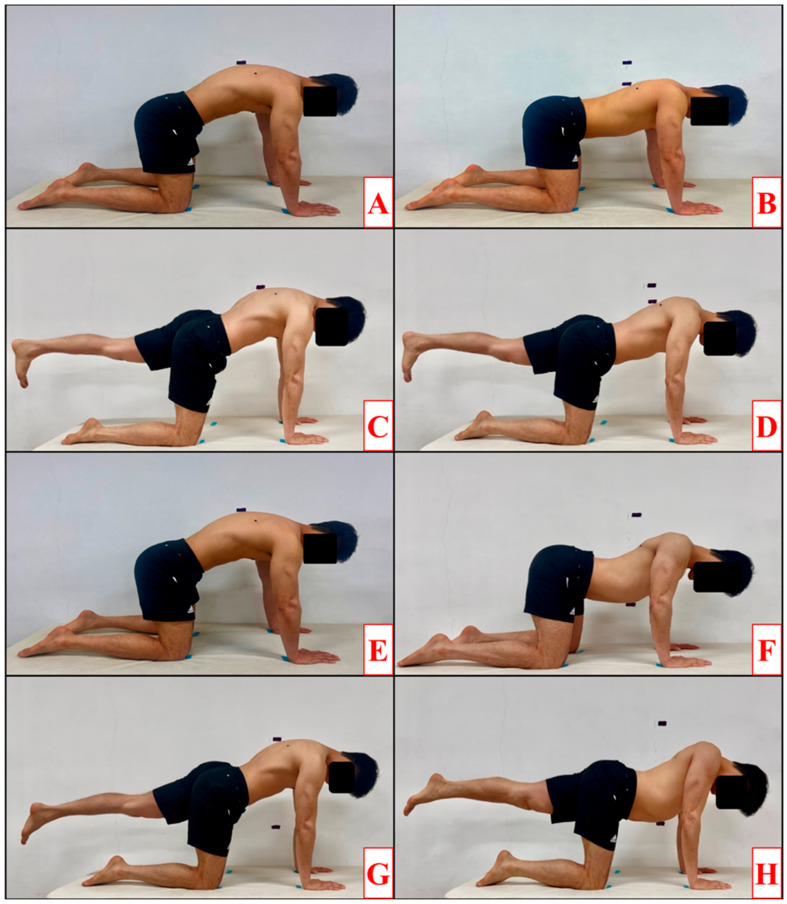
Protraction–Retraction Exercise Method During Four Variations (**A**,**B**) Push-up plus with quadruped; (**C**,**D**) Push-up plus with single-leg; (**E**,**F**) Sternum drop with quadruped; (**G**,**H**) Sternum drop with single-leg.

All exercises were performed in a quadruped position with approximately 90° of shoulder flexion and elbows extended, maintaining a neutral spine and pelvis throughout. The distance between hands and knees was standardized to one shoulder-to-hip length to ensure consistent loading. Participants were instructed to avoid trunk rotation and excessive scapular elevation during all phases. A total of four conditions of scapular protraction–retraction exercises were performed in the study. Each exercise consisted of three phases: scapular protraction, isometric contraction, and scapular retraction, each performed for 2 s. The speed of the phase was controlled to 2 s per stage using a metronome set to 60 beats per minute. All conditions were performed on a constant base of support, with hands and knees precisely aligned to floor markers [38]. Additional markers were placed at the sternum body and fifth thoracic spinous process positions at the end of the phase, and it was verified that the markers reached their designated positions at the end of each phase, ensuring accurate performance of each condition [39]. If the participant did not reach the designated marker during the exercise or did not maintain the set exercise speed, the trial was repeated. All exercise protocols are illustrated in Figure 1.

#### 2.4.1. Push-Up Plus with Quadruped (PUP_Q)

The PUP_Q exercise began in the quadruped position with the scapula in neutral position. During the protraction phase, the scapula was protracted to the maximum range of motion, and the position was maintained during the isometric contraction phase. The retraction phase returned the scapula to the neutral position.

#### 2.4.2. Sternum Drop with Quadruped (STD_Q)

The STD_Q was performed in the quadruped position with the scapula retraction to its maximum range of motion. During the scapula protraction phase, the scapula was protracted to its maximum range of motion, maintained in this position during the isometric contraction phase, and returned to the starting position during the scapula retraction phase.

#### 2.4.3. Single-Leg Condition

The single-leg condition consisted of push-ups plus with single-leg (PUP_S) and sternum drops with single-leg (STD_S). In this condition, the scapular protraction–retraction exercise was performed in a quadruped position with both hands and the non-dominant knee fixed on the floor, while the dominant leg was extended parallel to the ground [40]. Participants were instructed to keep the trunk aligned with the midline and avoid pelvic rotation throughout the single-leg tasks to minimize asymmetrical loading.

### 2.5. Outcome Measures

Surface electromyography (sEMG) activation was recorded using Acknowledge 5.0 software (version 5.0, BIOPAC Systems Inc., Goleta, CA, USA) with a six-channel BIOPAC MP160 device (BIOPAC Systems Inc., Goleta, CA, USA) (CMRR: >110 dB, impedance >10 MΩ, and band-pass filter: 20 Hz to 500 Hz). The sEMG signals were collected at a sampling rate of 2000 Hz. Prior to electrode placement, the skin was shaved, lightly abraded, and cleaned with alcohol to maintain impedance below 5 kΩ. Disposable bipolar Ag/AgCl gel electrodes (rectangular-type, diameter: 10 mm, interelectrode distance: 20 mm) were placed on six muscles (levator scapulae, pectoralis major, serratus anterior, upper trapezius, middle trapezius, and lower trapezius) according to SENIAM guidelines [37]. Surface electrodes were placed on the dominant side only to ensure consistent measurement and to minimize asymmetrical loading effects between sides during the single-leg condition. Ground electrodes were placed at the inferior angle of the scapula, olecranon, and acromion. Electrode positions are summarized in Table 1. To prevent analytical bias, all EMG data were numerically coded by an independent researcher, and the analyst responsible for EMG signal processing was blinded to the exercise condition labels during data cleaning and analysis.

The raw EMG signals were visually inspected for noise and DC offset, which was removed before processing. Filtered signals were processed using root mean square (RMS) values with a 50-ms moving window to balance signal smoothness and temporal resolution, as recommended by SENIAM and ISEK guidelines. All sEMG data were collected at the peak amplitudes during each phase and normalized to a percentage (%) using MVIC. The MVIC was assessed three times for each muscle, with each contraction held for 5 s and a 1-min rest between trials. Participants received standardized verbal encouragement and visual feedback to ensure maximal effort. All procedures followed SENIAM recommendations for joint stabilization and posture control, and detailed measurement methods are provided in Table 2 [41,42,43,44,45].

### 2.6. Statistical Analysis

All statistical analyses were performed using SPSS software (version 25.0, SPSS Inc., Chicago, IL, USA). The Shapiro–Wilk test was applied to verify the normality of the data [46]. A repeated-measures ANOVA was conducted to compare the four exercise conditions (PUP_Q, PUP_S, STD_Q, and STD_S). Bonferroni correction was applied for multiple comparisons, and effect sizes were expressed as partial eta squared (η^2^_p_) [47]. When Mauchly’s test revealed that the sphericity assumption was violated, appropriate correction factors were applied. Specifically, the Greenhouse–Geisser adjustment was used when the mean epsilon value was below 0.75, whereas the Huynh–Feldt adjustment was applied when it was 0.75 or higher [48,49,50]. Additionally, 95% confidence intervals were reported for the main comparisons to enhance interpretability and statistical transparency. The level of significance was set at α = 0.05.

## 3. Results

Twenty-six healthy male participants completed all experimental procedures. A total of six muscle activity analyses were performed, with four exercise conditions divided into three sections (scapular protraction, isometric, and scapular retraction phase).

### 3.1. Muscle Activation During the Scapular Protraction Phase

**Figure 2:**
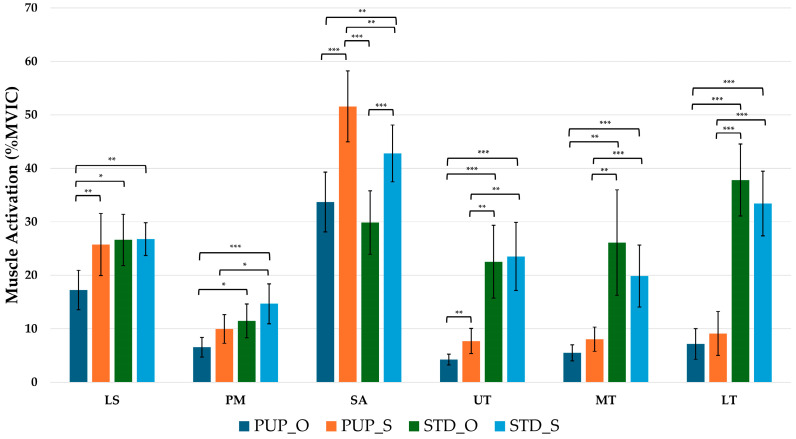
Post-hoc Muscle Activation (% MVIC) during the Scapular Protraction Phase. Bars represent the mean, and error bars indicate the 95% confidence interval. PUP_Q, push-up plus with quadruped; PUP_S, push-up plus with single-leg; STD_Q, sternum drop with quadruped; STD_S; LS, levator scapulae; PM, pectoralis major; SA, serratus anterior; UT, upper trapezius; MT, middle trapezius; LT, lower trapezius; * is significant difference, * = *p* < 0.05; ** is significant difference, ** = *p* < 0.01; *** is significant difference, *** = *p* < 0.001.

Significant differences with large effect sizes in all muscle activation were observed among the four exercise conditions during the scapular protraction phase. LS activation in PUP_Q was significantly lower than in all other conditions (*p* < 0.05). PM activation in PUP_Q was significantly lower than in STD_Q and STD_S (*p* < 0.001, *p* < 0.05, respectively), and PUP_S was significantly lower than in STD_S (*p* < 0.05). SA activation was highest in PUP_S, showing significantly greater activation than in all other conditions (*p* < 0.05). SA activation in STD_S showed the lowest activation, significantly different from all other conditions (*p* < 0.05). Upper trapezius (UT), middle trapezius (MT), and lower trapezius (LT) activations were significantly greater during both STD conditions compared with either push-up plus condition (*p* < 0.05). Only UT activation in PUP_Q was significantly different with PUP_S (*p* < 0.05). Scapular protraction muscle activation is shown in Table 3 and Figure 2.

### 3.2. Muscle Activation During the Scapular Isometric Phase

**Figure 3:**
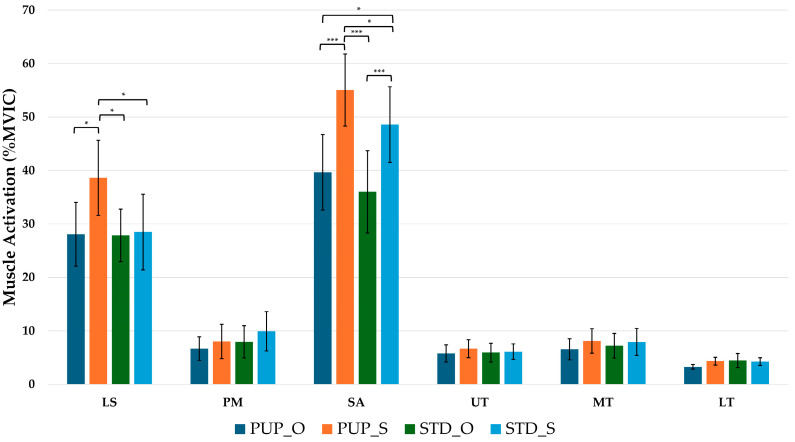
Post-hoc Muscle Activation (% MVIC) during the Scapular Isometric Phase. Bars represent the mean, and error bars indicate the 95% confidence interval. PUP_Q, push-up plus with quadruped; PUP_S, push-up plus with single-leg; STD_Q, sternum drop with quadruped; STD_S; LS, levator scapulae; PM, pectoralis major; SA, serratus anterior; UT, upper trapezius; MT, middle trapezius; LT, lower trapezius; * is significant difference, * = *p* < 0.05; *** is significant difference, *** = *p* < 0.001.

During the isometric phase, only LS and SA activation showed significant differences. LS showed significant differences (*p* = 0.001, F = 5.892, η^2^_p_ = 0.237), and the PUP_S condition was significantly greater than all other conditions (*p* < 0.05). SA activation in single-leg conditions was greater than quadruped conditions (*p* < 0.05), and the PUP_S condition was significantly greater than the STD_S condition (*p* < 0.05). Isometric phase muscle activation is presented in Table 4 and Figure 3.

### 3.3. Muscle Activation During the Scapular Retraction Phase

**Figure 4:**
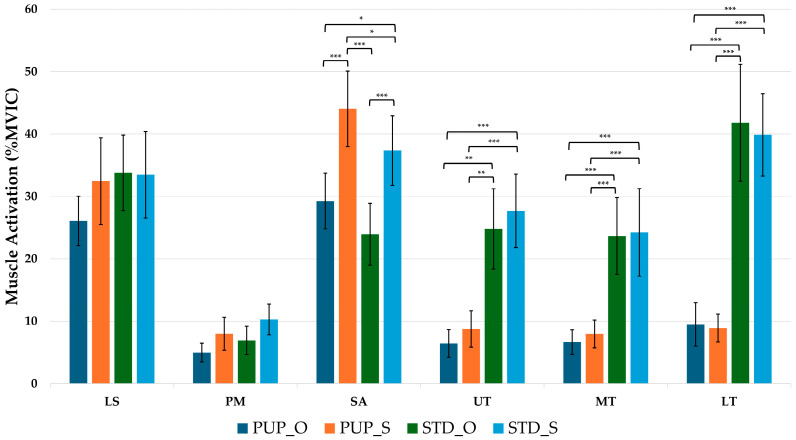
Post-hoc Muscle Activation (% MVIC) during the Scapular Retraction Phase. Bars represent the mean, and error bars indicate the 95% confidence interval. PUP_Q, push-up plus with quadruped; PUP_S, push-up plus with single-leg; STD_Q, sternum drop with quadruped; STD_S; LS, levator scapulae; PM, pectoralis major; SA, serratus anterior; UT, upper trapezius; MT, middle trapezius; LT, lower trapezius; * is significant difference, * = *p* < 0.05; ** is significant difference, ** = *p* < 0.01; *** is significant difference, *** = *p* < 0.001.

During the scapular retraction phase, all muscles except the LS showed statistically significant differences with large effect sizes. PM activation was significantly greater in STD_S compared with PUP_Q (*p* < 0.05). SA activation was significantly higher in PUP_S and STD_S compared with their quadruped positions (*p* < 0.05), and PUP_S also demonstrated greater activation than STD_S (*p* < 0.05). Trapezius activations were markedly higher in both STD conditions compared with either push-up plus condition (*p* < 0.05). Muscle activation during the scapular retraction phase is reported in Table 5 and Figure 4.

### 3.4. Scapular Muscle Activation Ratio

The UT/SA ratio was significantly lower in both push-up plus conditions than in the STD conditions (*p* < 0.001; η^2^_p_ = 0.544), while no significant difference was found in the UT/LT ratio among conditions (*p* = 0.287). Scapular muscle activation ratio is shown in Table 6.

## 4. Discussion

This study investigated differences in muscle activation during four variations of scapular protraction–retraction exercises across three movement phases. At the neuromuscular level, these patterns were identified through surface electromyography (EMG), allowing phase-specific interpretation of scapular muscle recruitment. The findings demonstrated distinct activation patterns according to exercise condition and phase, providing important implications for exercise prescription and rehabilitation protocols aimed at scapular stabilization. Moreover, in a closed-kinetic-chain environment, scapular stabilization reflects not only isolated muscular responses but also integrated neuromuscular control across the trunk–scapula–arm complex. Thus, the observed activation patterns likely represent alterations in coordinated motor control demands.

### 4.1. Scapular Protraction Phase

During the scapular protraction phase, SA activation in PUP_S was significantly higher by approximately 30% than in all other conditions. This enhanced activation may reflect the increased demand for scapular stabilization when the base of support is reduced by lifting one leg [40]. The serratus anterior is primarily responsible for scapular protraction and upward rotation [51], and its heightened activation in the single-leg push-up plus condition suggests that this exercise variant provides a superior training stimulus for this critical scapular stabilizer [52,53]. These results are consistent with prior studies reporting high SA activity during push-up plus exercises [40,54]. In a closed-kinetic-chain environment, the serratus anterior may function primarily to stabilize the thorax due to a relative reversal of its origin and insertion. The single-leg condition may increase rotational and postural demands, requiring greater SA activation to control thoracic motion and maintain trunk alignment [55].

Conversely, STD conditions demonstrated significantly greater activation of the trapezius muscles (upper, middle, and lower) compared to the push-up plus conditions. This pattern reflects the different starting positions and movement demands of these exercises [56,57]. Although research on the movement of the STD exercise is lacking, it is certainly an exercise that requires more scapular range of motion than PUP. In STD exercises, participants begin with scapular maximal retraction and move toward protraction, which may require greater trapezius activation to control the protraction phase and maintain scapular position throughout the movement [51,58].

Interestingly, levator scapulae (LS) activity in PUP_Q was significantly lower than in other conditions. This pattern is in line with previous work showing that certain push-up plus variants can reduce LS recruitment while emphasizing scapular upward rotators, which is desirable because excessive LS activation has been associated with maladaptive downward-rotation patterns and cervical/shoulder symptoms [59]. LS is believed to elevate, retract, and rotate the scapula downward [34]. The LS activation observed in PUP_Q compared to other conditions may indicate a more efficient movement pattern with reduced compensatory muscle activation [59]. Excessive levator scapulae activity is often associated with scapular dysfunction and neck pain [60,61,62], suggesting that PUP_Q may be a safer option for individuals with these concerns.

### 4.2. Isometric Phase

The isometric phase results observed differences in the importance of the base of support. Both single-leg conditions (PUP_S and STD_S) showed 20–25% significantly higher SA activation compared to their quadruped conditions. These results are consistent with previous studies, which have shown that instability caused by changing support may be accompanied by greater activation [12,25]. During the isometric phase, single-leg conditions increased SA activation by approximately 20–25% compared to the quadruped, indicating a clinically meaningful rise in stabilization demand. This predominance of SA activity during static control indicates that incorporating isometric hold components—particularly under reduced base-of-support conditions—may be relevant to early-phase scapular stabilization training. However, previous studies have reported inconsistent effects of support instability on SA activation, likely due to differences in load direction, trunk alignment, and exercise amplitude across protocols.

In particular, some authors found that instability did not increase SA recruitment when postural demand was low or when trunk motion was constrained, which may partially explain the contradictory findings [29,63,64]. In this study, the activity of SA was greatest in the isometric phase, unlike other muscles, which is similar to the results of Park and Yoo’s study [28], which also reported elevated SA activation during static protraction control; however, our results extend this evidence by showing that the single-leg condition further amplifies stabilization demand beyond traditional quadruped positions. This may support the need to include an isometric phase in PUPs and their variations to activate SA.

Interestingly, the levator scapulae showed similar patterns, with PUP_S demonstrating significantly greater activation than all other conditions. While this could indicate enhanced stabilization demands, further studies should monitor whether this increased activation represents functional stabilization or potentially problematic compensation patterns [5]. However, unlike the differences observed between PUP and STD in other phases, no significant changes were found in the remaining muscles (PM, UT, MT, LT). This suggests that these muscles maintain relatively consistent activation levels for stabilization during the isometric phase, regardless of exercise variations such as leg lifting [65]. Therefore, stabilizing activation may appear to depend more on sustained co-contraction of these muscles rather than on changes in the base of support during the isometric phase.

### 4.3. Scapular Retraction Phase

Consistent with other phases, the scapular retraction phase showed 15–20% higher SA activation in the single-leg conditions compared to quadrupedal conditions. These consistent patterns across all three phases support the use of single-leg variations rather than the quadruped variation to maximize SA activity. In the retraction phase, SA activation increased substantially under single-leg conditions, likely due to the greater thoracic stabilization demand in a closed-kinetic-chain environment, where the SA contributes to trunk rotation control as its functional origin–insertion relationship partially reverses.

Meanwhile, STD conditions maintained their advantage for trapezius muscle activation. This may be explained by the greater eccentric control and posterior-tilt demands imposed by the large scapular excursion in the STD movement. During the transition from maximal retraction to protraction, the upper, middle, and lower trapezius must stabilize upward rotation and posterior tilt, resulting in increased recruitment as ROM increases. These results suggest that the increased scapular range of motion from STD exercise did not affect SA activation. This appears to have influenced the trapezius group among other shoulder muscles [60,66]. Turgut et al. reported that stronger UT activation produces an increase in upward rotation [67]. This suggests that even when the STD exercise increases upward-rotation demand through larger scapular excursion, this may not necessarily lead to higher SA activation, as the UT can primarily compensate for the increased rotational requirement. While increased trapezius activation may be beneficial for individuals with weak scapular retractors [68], it could be counterproductive for those with already overactive upper trapezius muscles, a common pattern in individuals with forward head posture and rounded shoulders [69,70].

### 4.4. Stability Muscle Activation Ratio

The scapular muscle activity ratio analysis provides important clinical insight. Low UT/SA ratios observed in both push-up plus conditions suggest a more favorable stabilization pattern, consistent with prior evidence that excessive UT dominance is linked to scapular dyskinesis and shoulder pathology [64,71,72]. Accordingly, push-up plus variations may be preferable for individuals requiring enhanced SA activation while minimizing compensatory UT recruitment [12,26]. In contrast, the UT/LT ratio showed no significant difference across conditions, likely reflecting proportional co-activation rather than selective dominance.

Unlike previous studies that relied on summarized EMG values across the entire movement, this study provides phase-specific insight into protraction, isometric, and retraction demands. This methodological distinction allowed identification of activation patterns that would have been masked by traditional whole-movement analyses, offering a more precise understanding of scapular stabilization mechanisms.

### 4.5. Limitation of Study

This study has limitations. The sample consisted of trained young males, which limits generalizability. Surface EMG is susceptible to cross-talk and cannot assess deep muscles, and the absence of kinematic measures prevented confirmation of scapular motion. Although rest intervals were provided, fatigue effects cannot be fully excluded. EMG reflects activation rather than force or coordination, and individual variability was within expected ranges. Future studies should integrate EMG with kinematic or kinetic assessments to clarify mechanisms of scapular control. Additionally, the range of motion of the scapula could not be quantified due to individual differences in that part of the body.

## 5. Conclusions

This study examined scapular muscle activation across four protraction–retraction exercise variations, suggesting that modifications in the base of support and range of motion influence neuromuscular demands in healthy young males. Single-leg conditions showed greater serratus anterior activation compared with quadruped variations, whereas STD exercises produced higher trapezius recruitment. Push-up plus variations demonstrated lower UT/SA ratios, indicating a more favorable activation balance for scapular stabilization. These findings may provide useful guidance for rehabilitation planning. When the goal is to promote serratus anterior activation while minimizing compensatory upper trapezius activity, single-leg push-up plus variations may be considered. Conversely, STD variations may be appropriate for individuals requiring increased trapezius coactivation to improve controlled scapular retraction and posterior tilt. Additionally, incorporating isometric-hold components may benefit early-phase stabilization programs, given the relatively high serratus anterior activation observed during the isometric phase.

However, these conclusions should be interpreted with caution, as the sample consisted of trained healthy males and surface EMG reflected activation rather than strength or coordination. Future studies should integrate EMG with scapular kinematics or joint-stability analyses to better clarify the mechanisms underlying scapular control across exercise variations.

## Figures and Tables

**Table 1:** Surface Electromyography Electrode Location.

Muscle	Location
Levator scapulae	Lateral to the C3–C4 spinous process
Pectoralis major	Midclavicular line over the second intercostal space
Serratus anterior	6th to 8th rib space in the mid-axillary line
Upper trapezius	Midpoint between the acromion and the C7 spinous process
Middle trapezius	Midpoint between the T2 spinous process and scapular spine
Lower trapezius	Midpoint between the T7 spinous process and scapular spine

Note: All surface electrode positions for the six muscles were adopted from Hermens et al. (2000) [37].

**Table 2:** Maximal Voluntary Isometric Contraction Measurement Method.

Muscle	Initial Position	Isometric Contraction
Levator scapulae	sitting, head ipsilateral rotation, and shoulder elevation	shoulder elevation
Pectoralis major	supine, shoulder and elbow 90° flexion	shoulder horizontal adduction
Serratus anterior	sitting, shoulder 135° flexion	shoulder flexion
Upper trapezius	sitting, head contralateral rotation, lateral flexion, and shoulder elevation	head lateral flexion, shoulder elevation
Middle trapezius	prone, shoulder 90° abduction with thumb upward	scapular retraction
Lower trapezius	prone, shoulder 135° abduction with thumb upward	scapular retraction

Note: MVIC testing positions for the six muscles were adopted from McLean (2005) [41], Muyor et al. (2023) [42], Ekstrom et al. (2005) [43], Zanca et al. (2014) [44], and Kara & Harput (2019) [45].

**Table 3:** Muscle Activation (% MVIC) during the Scapular Protraction Phase.

Variable	PUP_Q (a)	PUP_S (b)	STD_Q (c)	STD_S (d)	*p*-Value	F	η^2^_p_
LS (%)	17.25 ± 7.84 ^b,c,d^	25.76 ± 12.41 ^a^	26.62 ± 10.24 ^a^	26.75 ± 6.54 ^a^	0.011 *	5.728	0.232
PM (%)	6.55 ± 4.22 ^c,d^	9.95 ± 6.21 ^d^	11.46 ± 7.30 ^a^	14.66 ± 8.64 ^a,b^	<0.001 **	10.279	0.318
SA (%)	33.70 ± 13.27 ^b,d^	51.57 ± 15.72 ^a,c,d^	29.85 ± 14.04 ^b,d^	24.79 ± 12.59 ^a,b,c^	<0.001 **	41.679	0.644
UT (%)	4.25 ± 2.18 ^b,c,d^	7.68 ± 5.16 ^a,c,d^	22.56 ± 14.96 ^a,b^	23.51 ± 13.98 ^a,b^	<0.001 **	22.648	0.531
MT (%)	5.49 ± 3.30 ^c,d^	8.04 ± 4.97 ^c,d^	26.11 ± 21.68 ^a,b^	19.87 ± 12.76 ^a,b^	<0.001 **	19.193	0.490
LT (%)	7.14 ± 6.47 ^c,d^	9.12 ± 9.28 ^c,d^	37.81 ± 15.18 ^a,b^	33.42 ± 13.61 ^a,b^	<0.001 **	47.433	0.693

Data are expressed as the mean ± standard deviation. PUP_Q, push-up plus with quadruped; PUP_S, push-up plus with single-leg; STD_Q, sternum drop with quadruped; STD_S, sternum drop with single-leg; η^2^_p_, partial eta squared; LS, levator scapulae; PM, pectoralis major; SA, serratus anterior; UT, upper trapezius; MT, middle trapezius; LT, lower trapezius. ^a^ is significant difference from PUP_Q (*p* < 0.05). ^b^ is significant difference from PUP_S (*p* < 0.05). ^c^ is significant difference from STD_Q (*p* < 0.05). ^d^ is significant difference from STD_S (*p* < 0.05). * is significant difference, * = *p* < 0.05. ** is significant difference, ** = *p* < 0.001.

**Table 4:** Muscle Activation (% MVIC) during the Isometric Phase.

Variable	PUP_Q (a)	PUP_S (b)	STD_Q (c)	STD_S (d)	*p*-Value	F	η^2^_p_
LS (%)	28.07 ± 12.75 ^b^	38.63 ± 14.99 ^a,c,d^	27.86 ± 10.47 ^b^	28.49 ± 15.11 ^b^	0.001 *	5.892	0.237
PM (%)	6.66 ± 4.82	8.02 ± 7.04	7.96 ± 6.63	9.92 ± 8.04	0.102	2.157	0.097
SA (%)	39.66 ± 16.36 ^b,d^	55.06 ± 15.59 ^a,c,d^	36.00 ± 17.83 ^b,d^	48.58 ± 16.37 ^a,b,c^	<0.001 **	24.775	0.530
UT (%)	5.76 ± 3.41	6.66 ± 3.57	5.92 ± 3.78	6.11 ± 3.10	0.458	0.877	0.044
MT (%)	6.56 ± 4.30	8.10 ± 5.04	7.22 ± 5.05	7.92 ± 5.55	0.492	0.812	0.039
LT (%)	3.26 ± 0.97	4.33 ± 1.59	4.44 ± 2.85	4.24 ± 1.59	0.095	2.671	0.118

Data are expressed as the mean ± standard deviation. PUP_Q, push-up plus with quadruped; PUP_S, push-up plus with single-leg; STD_Q, sternum drop with quadruped; STD_S, sternum drop with single-leg; η^2^_p_, partial eta squared; LS, levator scapulae; PM, pectoralis major; SA, serratus anterior; UT, upper trapezius; MT, middle trapezius; LT, lower trapezius. ^a^ is significant difference from PUP_Q (*p* < 0.05). ^b^ is significant difference from PUP_S (*p* < 0.05). ^c^ is significant difference from STD_Q (*p* < 0.05). ^d^ is significant difference from STD_S (*p* < 0.05). * is significant difference, * = *p* < 0.05. ** is significant difference, ** = *p* < 0.001.

**Table 5:** Muscle Activation (% MVIC) during the Scapular Retraction Phase.

Variable	PUP_Q (a)	PUP_S (b)	STD_Q (c)	STD_S (d)	*p*-Value	F	η^2^_p_
LS (%)	26.07 ± 8.71	32.45 ± 15.27	33.77 ± 13.29	33.47 ± 15.24	0.113	2.134	0.096
PM (%)	4.98 ± 3.30 ^d^	7.97 ± 5.80	6.94 ± 4.97	10.28 ± 5.43 ^a^	0.001 *	6.558	0.247
SA (%)	29.25 ± 10.05 ^b,d^	44.03 ± 13.64 ^a,c,d^	23.95 ± 11.16 ^b,d^	37.36 ± 12.60 ^a,b,c^	<0.001 **	25.942	0.553
UT (%)	6.43 ± 4.91 ^c,d^	8.76 ± 6.37 ^c,d^	24.79 ± 14.14 ^a,b^	27.68 ± 12.92 ^a,b^	<0.001 **	27.797	0.582
MT (%)	6.66 ± 4.35 ^c,d^	7.97 ± 4.87 ^c,d^	23.67 ± 13.48 ^a,b^	24.24 ± 15.40 ^a,b^	<0.001 **	24.841	0.554
LT (%)	9.50 ± 8.03 ^c,d^	8.91 ± 5.20 ^c,d^	41.81 ± 21.67 ^a,b^	39.86 ± 15.25 ^a,b^	<0.001 **	48.987	0.690

Data are expressed as the mean ± standard deviation. PUP_Q, push-up plus with quadruped; PUP_S, push-up plus with single-leg; STD_Q, sternum drop with quadruped; STD_S, sternum drop with single-leg; η^2^_p_, partial eta squared; LS, levator scapulae; PM, pectoralis major; SA, serratus anterior; UT, upper trapezius; MT, middle trapezius; LT, lower trapezius. ^a^ is significant difference from PUP_Q (*p* < 0.05). ^b^ is significant difference from PUP_S (*p* < 0.05). ^c^ is significant difference from STD_Q (*p* < 0.05). ^d^ is significant difference from STD_S (*p* < 0.05). * is significant difference, * = *p* < 0.05. ** is significant difference, ** = *p* < 0.001. We have added Figure 4, and it is now mentioned in the text immediately above Table 5.

**Table 6:** Stability Muscle Activation Ratio.

Variable	PUP_Q (a)	PUP_S (b)	STD_Q (c)	STD_S (d)	*p*-Value	F	η^2^_p_
UT/SA	0.18 ± 0.13 ^c,d^	0.18 ± 0.17 ^c,d^	0.70 ± 0.44 ^a,b^	0.47 ± 0.21 ^a,b^	<0.001 **	23.901	0.544
UT/LT	1.45 ± 1.18	1.57 ± 1.56	1.25 ± 1.05	1.22 ± 0.90	0.287	1.292	0.064

Data are expressed as the mean ± standard deviation. PUP_Q, push-up plus with quadruped; PUP_S, push-up plus with single-leg; STD_Q, sternum drop with quadruped; STD_S, sternum drop with single-leg; η^2^_p_, partial eta squared; LS, levator scapulae; PM, pectoralis major; SA, serratus anterior; UT, upper trapezius; MT, middle trapezius; LT, lower trapezius. ^a^ is significant difference from PUP_Q (*p* < 0.05). ^b^ is significant difference from PUP_S (*p* < 0.05). ^c^ is significant difference from STD_Q (*p* < 0.05). ^d^ is significant difference from STD_S (*p* < 0.05). ** is significant difference, ** = *p* < 0.001.

## Data Availability

The data presented in this study are available on request from the corresponding author. The data are not publicly available due to ethical restrictions.
